# [(*E*)-But-2-enoato-κ*O*]chlorido(2,2′-diamino-4,4′-bi-1,3-thia­zole-κ^2^ *N* ^3^,*N* ^3′^)zinc(II) monohydrate

**DOI:** 10.1107/S160053681001113X

**Published:** 2010-03-27

**Authors:** Mei Du, Yan-Li Wang, Bing-Xin Liu, Duan-Jun Xu

**Affiliations:** a Department of Chemistry, Shanghai University, People’s Republic of China; b Department of Chemistry, Zhejiang University, People’s Republic of China

## Abstract

In the title compound, [Zn(C_4_H_5_O_2_)Cl(C_6_H_6_N_4_S_2_)]·H_2_O, the Zn^II^ cation is coordinated by a bidentate diamino­bithia­zole (DABT) ligand, a but-2-enoate anion and a Cl^−^ anion in a distorted tetra­hedral geometry. Within the DABT ligand, the two thia­zole rings are twisted to each other at a dihedral angle of 4.38 (10)°. An intra­molecular N—H⋯O inter­action occurs. The centroid–centroid distance of 3.6650 (17) Å and partially overlapped arrangement between nearly parallel thia­zole rings of adjacent complexes indicate the existence of π–π stacking in the crystal structure. Extensive O—H⋯Cl, O—H⋯O, N—H⋯Cl and N—H⋯O hydrogen bonding helps to stabilize the crystal structure.

## Related literature

For the potential applications of metal complexes of diamino­bithia­zole in the biological field, see: Waring (1981 ▶); Fisher *et al.* (1985 ▶). For dihedral angles between thia­zole rings in diamino­bithia­zole complexes, see: Du *et al.* (2010 ▶); Zhang *et al.* (2006 ▶).
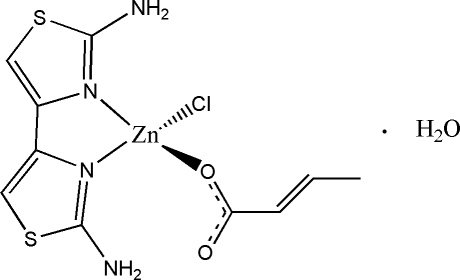


## Experimental

### 

#### Crystal data

[Zn(C_4_H_5_O_2_)Cl(C_6_H_6_N_4_S_2_)]·H_2_O
*M* *_r_* = 402.18
Monoclinic, 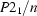

*a* = 7.2782 (13) Å
*b* = 16.2846 (16) Å
*c* = 13.237 (2) Å
β = 99.252 (16)°
*V* = 1548.5 (4) Å^3^
*Z* = 4
Mo *K*α radiation
μ = 2.04 mm^−1^
*T* = 294 K
0.36 × 0.30 × 0.24 mm

#### Data collection

Rigaku R-AXIS RAPID IP diffractometer
Absorption correction: multi-scan (*ABSCOR*; Higashi, 1995 ▶) *T*_min_ = 0.75, *T*_max_ = 0.88
7862 measured reflections
2735 independent reflections
2293 reflections with *I* > 2σ(*I*)
*R*_int_ = 0.026

#### Refinement

*R*[*F* ^2^ > 2σ(*F* ^2^)] = 0.032
*wR*(*F* ^2^) = 0.080
*S* = 1.05
2735 reflections
191 parameters
H-atom parameters constrained
Δρ_max_ = 0.71 e Å^−3^
Δρ_min_ = −0.45 e Å^−3^

### 

Data collection: *PROCESS-AUTO* (Rigaku, 1998 ▶); cell refinement: *PROCESS-AUTO*; data reduction: *CrystalStructure* (Rigaku/MSC, 2002 ▶); program(s) used to solve structure: *SIR92* (Altomare *et al.*, 1993 ▶); program(s) used to refine structure: *SHELXL97* (Sheldrick, 2008 ▶); molecular graphics: *ORTEP-3 for Windows* (Farrugia, 1997 ▶); software used to prepare material for publication: *WinGX* (Farrugia, 1999 ▶).

## Supplementary Material

Crystal structure: contains datablocks I, global. DOI: 10.1107/S160053681001113X/ng2750sup1.cif

Structure factors: contains datablocks I. DOI: 10.1107/S160053681001113X/ng2750Isup2.hkl

Additional supplementary materials:  crystallographic information; 3D view; checkCIF report

## Figures and Tables

**Table 1:** Selected bond lengths (Å)

Zn—O1	1.961 (2)
Zn—N1	2.029 (2)
Zn—N3	2.060 (2)
Zn—Cl1	2.2223 (9)

**Table 2:** Hydrogen-bond geometry (Å, °)

*D*—H⋯*A*	*D*—H	H⋯*A*	*D*⋯*A*	*D*—H⋯*A*
O1*W*—H1*A*⋯Cl1^i^	0.86	2.56	3.345 (3)	152
O1*W*—H1*B*⋯O1	0.86	2.04	2.859 (4)	160
N2—H2*A*⋯O2	0.86	2.22	2.959 (4)	144
N2—H2*B*⋯O1*W*^ii^	0.86	2.23	3.032 (4)	154
N4—H4*A*⋯O1*W*	0.86	2.30	3.043 (4)	145
N4—H4*B*⋯Cl1^iii^	0.86	2.66	3.393 (3)	144

Symmetry codes: (i) 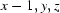; (ii) 
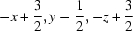
; (iii) 
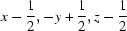
.

**Fig. 1.**
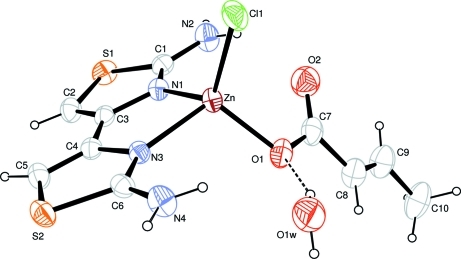
The molecular structure of the title compound with 40% probability displacement ellipsoids. Dashed lines indicate the hydrogen bonding.

**Fig. 2.**
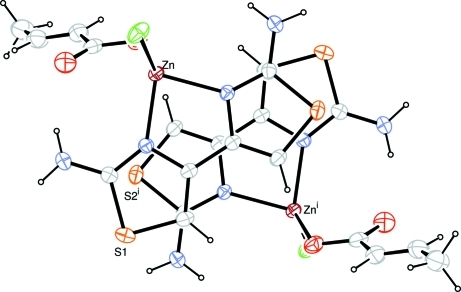
The partially overlapped arrangement between thiazole rings showing π-π stacking [symmetry code: (i) 1-x, -y, 1-z].

## supplementary crystallographic information

### Comment

Some metal complexes with 2,2'-diamino-4,4'-bi-1,3-thiazole (DABT) have shown the potential application in the biological field (Waring, 1981; Fisher *et al.*, 1985). As a part of serial structural investigation of metal complexes with DABT, the title Zn^II^ complex was prepared in the laboratory and its X-ray structure is presented here.

The molecular structure of the title compound is shown in Fig. 1. The Zn^II^ cation is coordinated by a diaminobithiazole (DABT) ligand, a but-2-enoate anion and a Cl^-^ anion in a distorted tetrahedral geometry (Table 1). Within the DABT ligand the two thiazole rings are twisted to each other at a dihedral angle of 4.38 (10)°, which agrees with 9.51 (17)° found in a Pb^II^ complex of DABT (Du *et al.*, 2010) and 9.5 (2)° found in a Cd^II^ complex of DABT (Zhang *et al.*, 2006). The partially overlapped arrangement of centroids distance of 3.6650 (17) Å between nearly parallel thiazole rings of the adjacent complexes indicate the existence of π-π stacking in the crystal structure (Fig. 2). The extensive hydrogen bonding help to stabilize the crystal structure (Table 2).

### Experimental

A water-ethanol solution (20 ml, 1:1) of DABT (0.10 g, 0.5 mmol) and ZnCl_2_ (0.07 g, 0.5 mmol) was refluxed for 10 min, then an aqueous solution (20 ml) of (E)-but-2-enoatic acid (0.09 g, 1 mmol) and NaOH (0.04 g, 1 mmol) was mixed with the above solution. The mixture was refluxed for 6 h and then filtered. The single crystals of the title compound were obtained from the filtrate after a week.

### Refinement

H atoms of water molecule were located in a difference Fourier map and were refined as riding in as-found relative positions with U_iso_(H) = 1.2U_eq_(O). Other H atoms were placed in calculated positions with C—H = 0.96 Å (methyl), 0.93 Å (aromatic) and N—H = 0.86 Å, and refined in the riding model with U_iso_(H) = 1.5U_eq_(C) for methyl and 1.2U_eq_(C,*N*) for the others.

### Crystal data

**Table:**

[Zn(C_4_H_5_O_2_)Cl(C_6_H_6_N_4_S_2_)]·H_2_O	*F*(000) = 816
*M**_r_* = 402.18	*D*_x_ = 1.725 Mg m^−^^3^
Monoclinic, *P*2_1_/*n*	Mo *K*α radiation, λ = 0.71073 Å
Hall symbol: -P 2yn	Cell parameters from 3446 reflections
*a* = 7.2782 (13) Å	θ = 2.0–24.6°
*b* = 16.2846 (16) Å	µ = 2.04 mm^−^^1^
*c* = 13.237 (2) Å	*T* = 294 K
β = 99.252 (16)°	Block, yellow
*V* = 1548.5 (4) Å^3^	0.36 × 0.30 × 0.24 mm
*Z* = 4	

### Data collection

**Table:**

Rigaku R-AXIS RAPID IP diffractometer	2735 independent reflections
Radiation source: fine-focus sealed tube	2293 reflections with *I* > 2σ(*I*)
graphite	*R*_int_ = 0.026
ω scans	θ_max_ = 25.0°, θ_min_ = 2.0°
Absorption correction: multi-scan (*ABSCOR*; Higashi, 1995)	*h* = −8→8
*T*_min_ = 0.75, *T*_max_ = 0.88	*k* = −12→19
7862 measured reflections	*l* = −15→15

### Refinement

**Table:**

Refinement on *F*^2^	Primary atom site location: structure-invariant direct methods
Least-squares matrix: full	Secondary atom site location: difference Fourier map
*R*[*F*^2^ > 2σ(*F*^2^)] = 0.032	Hydrogen site location: inferred from neighbouring sites
*wR*(*F*^2^) = 0.080	H-atom parameters constrained
*S* = 1.05	*w* = 1/[σ^2^(*F*_o_^2^) + (0.0353*P*)^2^ + 1.093*P*] where *P* = (*F*_o_^2^ + 2*F*_c_^2^)/3
2735 reflections	(Δ/σ)_max_ = 0.002
191 parameters	Δρ_max_ = 0.71 e Å^−^^3^
0 restraints	Δρ_min_ = −0.45 e Å^−^^3^

### Special details

**Table:**

Geometry. All esds (except the esd in the dihedral angle between two l.s. planes) are estimated using the full covariance matrix. The cell esds are taken into account individually in the estimation of esds in distances, angles and torsion angles; correlations between esds in cell parameters are only used when they are defined by crystal symmetry. An approximate (isotropic) treatment of cell esds is used for estimating esds involving l.s. planes.
Refinement. Refinement of *F*^2^ against ALL reflections. The weighted *R*-factor wR and goodness of fit *S* are based on *F*^2^, conventional *R*-factors *R* are based on *F*, with *F* set to zero for negative *F*^2^. The threshold expression of *F*^2^ > σ(*F*^2^) is used only for calculating *R*-factors(gt) etc. and is not relevant to the choice of reflections for refinement. *R*-factors based on *F*^2^ are statistically about twice as large as those based on *F*, and *R*- factors based on ALL data will be even larger.

### Fractional atomic coordinates and isotropic or equivalent isotropic displacement parameters (Å^2^)

**Table:**

	*x*	*y*	*z*	*U*_iso_*/*U*_eq_	
Zn	0.77894 (5)	0.12724 (2)	0.64617 (2)	0.03260 (13)	
Cl1	1.01129 (13)	0.21725 (6)	0.67179 (7)	0.0541 (3)	
S1	0.93933 (12)	−0.14538 (5)	0.65212 (7)	0.0433 (2)	
S2	0.56191 (12)	0.09881 (5)	0.30033 (6)	0.0418 (2)	
N1	0.8456 (3)	0.00649 (15)	0.64140 (17)	0.0314 (6)	
N2	0.9835 (4)	−0.03495 (18)	0.8057 (2)	0.0483 (7)	
H2A	0.9737	0.0134	0.8305	0.058*	
H2B	1.0326	−0.0740	0.8447	0.058*	
N3	0.6716 (3)	0.10813 (15)	0.49432 (17)	0.0299 (5)	
N4	0.5365 (4)	0.22969 (16)	0.4215 (2)	0.0444 (7)	
H4A	0.5508	0.2545	0.4796	0.053*	
H4B	0.4859	0.2549	0.3670	0.053*	
O1	0.5737 (3)	0.15367 (14)	0.72007 (17)	0.0471 (6)	
O2	0.7729 (4)	0.11162 (17)	0.8535 (2)	0.0630 (7)	
O1W	0.4071 (4)	0.29467 (17)	0.6138 (2)	0.0726 (8)	
H1A	0.2909	0.2835	0.6079	0.087*	
H1B	0.4522	0.2592	0.6587	0.087*	
C1	0.9224 (4)	−0.04933 (19)	0.7066 (2)	0.0351 (7)	
C2	0.8373 (4)	−0.10659 (19)	0.5354 (2)	0.0396 (8)	
H2	0.8137	−0.1368	0.4751	0.047*	
C3	0.7965 (4)	−0.02684 (18)	0.5437 (2)	0.0316 (7)	
C4	0.7091 (4)	0.02855 (18)	0.4637 (2)	0.0309 (7)	
C5	0.6607 (4)	0.0136 (2)	0.3637 (2)	0.0386 (7)	
H5	0.6782	−0.0363	0.3325	0.046*	
C6	0.5924 (4)	0.15275 (19)	0.4159 (2)	0.0333 (7)	
C7	0.6148 (5)	0.1325 (2)	0.8138 (3)	0.0448 (8)	
C8	0.4511 (6)	0.1316 (2)	0.8698 (3)	0.0541 (9)	
H8	0.3377	0.1509	0.8357	0.065*	
C9	0.4583 (6)	0.1061 (2)	0.9613 (3)	0.0623 (11)	
H9	0.5745	0.0909	0.9963	0.075*	
C10	0.2972 (7)	0.0984 (3)	1.0172 (3)	0.0724 (13)	
H10A	0.3061	0.1397	1.0696	0.109*	
H10B	0.2981	0.0450	1.0479	0.109*	
H10C	0.1834	0.1057	0.9702	0.109*	

### Atomic displacement parameters (Å^2^)

**Table:**

	*U*^11^	*U*^22^	*U*^33^	*U*^12^	*U*^13^	*U*^23^
Zn	0.0384 (2)	0.0298 (2)	0.0298 (2)	−0.00060 (15)	0.00584 (14)	−0.00417 (15)
Cl1	0.0597 (6)	0.0545 (6)	0.0507 (5)	−0.0236 (4)	0.0161 (4)	−0.0186 (4)
S1	0.0460 (5)	0.0295 (4)	0.0551 (5)	0.0035 (4)	0.0103 (4)	0.0042 (4)
S2	0.0460 (5)	0.0489 (5)	0.0286 (4)	−0.0057 (4)	0.0004 (3)	−0.0015 (4)
N1	0.0353 (14)	0.0292 (13)	0.0305 (13)	−0.0011 (11)	0.0076 (10)	0.0008 (11)
N2	0.0629 (19)	0.0439 (17)	0.0357 (15)	0.0110 (14)	0.0010 (13)	0.0064 (13)
N3	0.0316 (13)	0.0310 (13)	0.0279 (12)	−0.0041 (11)	0.0069 (10)	−0.0008 (10)
N4	0.0572 (18)	0.0363 (16)	0.0371 (15)	0.0029 (13)	0.0001 (13)	0.0059 (12)
O1	0.0528 (15)	0.0469 (14)	0.0449 (14)	0.0028 (11)	0.0173 (11)	−0.0083 (11)
O2	0.0653 (19)	0.0609 (17)	0.0627 (17)	0.0061 (14)	0.0103 (14)	−0.0015 (14)
O1W	0.0642 (18)	0.072 (2)	0.084 (2)	−0.0139 (15)	0.0188 (15)	−0.0228 (16)
C1	0.0323 (17)	0.0348 (17)	0.0397 (18)	−0.0008 (14)	0.0100 (13)	0.0035 (14)
C2	0.046 (2)	0.0319 (18)	0.0428 (18)	−0.0028 (14)	0.0115 (15)	−0.0064 (14)
C3	0.0298 (16)	0.0315 (17)	0.0353 (16)	−0.0052 (13)	0.0110 (12)	−0.0043 (13)
C4	0.0310 (16)	0.0304 (16)	0.0329 (16)	−0.0055 (13)	0.0098 (12)	−0.0025 (13)
C5	0.0449 (19)	0.0372 (18)	0.0343 (17)	−0.0060 (15)	0.0081 (14)	−0.0069 (14)
C6	0.0337 (17)	0.0347 (17)	0.0309 (16)	−0.0053 (13)	0.0038 (13)	0.0006 (13)
C7	0.056 (2)	0.0315 (18)	0.050 (2)	−0.0028 (16)	0.0166 (17)	−0.0093 (16)
C8	0.062 (2)	0.051 (2)	0.051 (2)	0.0083 (18)	0.0151 (18)	−0.0034 (18)
C9	0.079 (3)	0.050 (2)	0.062 (3)	0.014 (2)	0.022 (2)	0.002 (2)
C10	0.097 (3)	0.063 (3)	0.069 (3)	0.017 (2)	0.048 (3)	0.011 (2)

### Geometric parameters (Å, °)

**Table:**

Zn—O1	1.961 (2)	O1—C7	1.275 (4)
Zn—N1	2.029 (2)	O2—C7	1.234 (4)
Zn—N3	2.060 (2)	O1W—H1A	0.8564
Zn—Cl1	2.2223 (9)	O1W—H1B	0.8550
S1—C2	1.723 (3)	C2—C3	1.340 (4)
S1—C1	1.735 (3)	C2—H2	0.9300
S2—C5	1.718 (3)	C3—C4	1.457 (4)
S2—C6	1.747 (3)	C4—C5	1.336 (4)
N1—C1	1.315 (4)	C5—H5	0.9300
N1—C3	1.395 (4)	C7—C8	1.502 (5)
N2—C1	1.336 (4)	C8—C9	1.273 (5)
N2—H2A	0.8600	C8—H8	0.9300
N2—H2B	0.8600	C9—C10	1.490 (5)
N3—C6	1.321 (4)	C9—H9	0.9300
N3—C4	1.398 (4)	C10—H10A	0.9600
N4—C6	1.323 (4)	C10—H10B	0.9600
N4—H4A	0.8600	C10—H10C	0.9600
N4—H4B	0.8600		
			
O1—Zn—N1	115.69 (10)	C2—C3—N1	115.2 (3)
O1—Zn—N3	108.68 (10)	C2—C3—C4	128.1 (3)
N1—Zn—N3	83.01 (9)	N1—C3—C4	116.7 (2)
O1—Zn—Cl1	113.65 (7)	C5—C4—N3	115.0 (3)
N1—Zn—Cl1	117.66 (7)	C5—C4—C3	128.5 (3)
N3—Zn—Cl1	114.11 (7)	N3—C4—C3	116.5 (2)
C2—S1—C1	89.60 (15)	C4—C5—S2	111.1 (2)
C5—S2—C6	89.66 (15)	C4—C5—H5	124.5
C1—N1—C3	111.0 (2)	S2—C5—H5	124.5
C1—N1—Zn	136.7 (2)	N3—C6—N4	125.2 (3)
C3—N1—Zn	112.28 (18)	N3—C6—S2	112.9 (2)
C1—N2—H2A	120.0	N4—C6—S2	121.9 (2)
C1—N2—H2B	120.0	O2—C7—O1	123.1 (3)
H2A—N2—H2B	120.0	O2—C7—C8	123.1 (3)
C6—N3—C4	111.3 (2)	O1—C7—C8	113.7 (3)
C6—N3—Zn	137.1 (2)	C9—C8—C7	124.0 (4)
C4—N3—Zn	111.23 (18)	C9—C8—H8	118.0
C6—N4—H4A	120.0	C7—C8—H8	118.0
C6—N4—H4B	120.0	C8—C9—C10	125.8 (4)
H4A—N4—H4B	120.0	C8—C9—H9	117.1
C7—O1—Zn	110.3 (2)	C10—C9—H9	117.1
H1A—O1W—H1B	100.6	C9—C10—H10A	109.5
N1—C1—N2	124.2 (3)	C9—C10—H10B	109.5
N1—C1—S1	113.6 (2)	H10A—C10—H10B	109.5
N2—C1—S1	122.1 (2)	C9—C10—H10C	109.5
C3—C2—S1	110.6 (2)	H10A—C10—H10C	109.5
C3—C2—H2	124.7	H10B—C10—H10C	109.5
S1—C2—H2	124.7		

### Hydrogen-bond geometry (Å, °)

**Table:**

*D*—H···*A*	*D*—H	H···*A*	*D*···*A*	*D*—H···*A*
O1W—H1A···Cl1^i^	0.86	2.56	3.345 (3)	152
O1W—H1B···O1	0.86	2.04	2.859 (4)	160
N2—H2A···O2	0.86	2.22	2.959 (4)	144
N2—H2B···O1W^ii^	0.86	2.23	3.032 (4)	154
N4—H4A···O1W	0.86	2.30	3.043 (4)	145
N4—H4B···Cl1^iii^	0.86	2.66	3.393 (3)	144

Symmetry codes: (i) *x*−1, *y*, *z*; (ii) −*x*+3/2, *y*−1/2, −*z*+3/2; (iii) *x*−1/2, −*y*+1/2, *z*−1/2.
